# Establishment and phenotyping of neurosphere cultures from primary neuroblastoma samples

**DOI:** 10.12688/f1000research.18209.1

**Published:** 2019-06-10

**Authors:** Jack Barton, Katherine Pacey, Neha Jain, Tessa Kasia, Darren Edwards, Christine Thevanesan, Karin Straathof, Giuseppe Barone, John Anderson

**Affiliations:** 1 Cancer Section, DBC Programme, UCL Great Ormond Street Institute of Child Health, London, UK; 2 Oncology, Great Ormond Street Hospital for Children, London, UK

**Keywords:** neuroblastoma, neurosphere, stem cell

## Abstract

**Background**: Primary cell culture using serum free media supplemented with growth factors has been used in a number of cancers to propagate primary cells with stem like properties, which form as spherical cellular aggregates.

**Methods**: We systematically evaluated the capacity of freshly disaggregated neuroblastoma tumors to become established as neurospheres in stem cell media using a uniform protocol. 67 primary neuroblastoma samples from patients treated at a single institution were prospectively evaluated for their ability to become established in culture. Samples, either solid tissue or cells from surgical transit fluid both post chemotherapy and chemotherapy naïve, were evaluated from diagnostic needle biopsies or surgical resections.

**Results**: Overall 37 neurosphere cultures were successfully established from 67 samples. In 11 out of 14 cases investigated by flow cytometry, uniform staining for neuroblastoma markers CD56 and GD2 was demonstrated in CD45 negative non-hemopoietic cells, confirming neuroblastoma origin.

**Conclusion**: We present a simple and reproducible approach for producing primary neurospheres from neuroblastoma samples, which provides a reliable resource for future work including genetic analysis, stem cell research and models for therapeutics.

**Table:** 

Abbreviation	Full term
NBL	Neuroblastoma
SCM	Stem Cell Media
CNS	Central Nervous System
EGF	Epidermal Growth Factor
FGF	Fibroblast Growth Factor

## Background

Neuroblastoma is the most common extra-cranial solid tumor occurring in children, and the most commonly diagnosed cancer in children under the age of 1 year ^1^. Tumors are thought to have embryological origin in the neural crest, arise within the sympathetic chain or adrenal gland, and are frequently metastatic ^2^. The disease is strikingly heterogeneous with a range of clinical phenotypes, from spontaneous regression, including in cases with metastases, to aggressive disease with marked resistance to radiotherapy and chemotherapy. New approaches to therapies supported by the availability of suitable tumor models that more closely resemble the human disease are essential for improving outcomes for this disease.

Established neuroblastoma cell lines have the advantage of being widely available, and enable comparison of research worldwide by their use as a common standard. However, cell lines are limited in their ability to reflect disease physiology due to adaptation to prolonged culturing conditions. Inevitable evolution and changes in transcriptional response occur during culturing, which vary between laboratories ^3, 4^, and may alter diverse cell behaviors such as drug response. The potential for proliferation of subclones with greater intrinsic capacity to survive *in vitro* may lead to loss of genetic variability within a cell line compared with polyclonal cancer populations in humans.

Increasingly it is recognized that *in vivo* mouse models of tumors, established in immunodeficient animals following minimal or no *in vitro* passaging (Patient-Derived Xenografts; PDX) is a valuable tool for evaluation of therapeutic agents. Whilst many PDX models have been established by immediate surgical implantation of freshly acquired tumor surgical samples, this is not always a practical approach. Neurospheres are spherical cellular clusters derived from neural stem cells, that develop under culture conditions of serum free media supplemented with growth factors ^5^. Initially produced from adult CNS tissue, they were the first demonstration of proliferative capacity in the adult brain, and have since been derived from embryonic stem cells, and CNS tumor cells. Cancer stem cells have been identified in a range of malignancies ^6^, and have been implicated in initiation and progression of solid tumors ^7^, and development of resistance to therapy. Growth of neurospheres in stem cell media is hypothesized to enrich for cancer stem cells. Given the importance of cancer stem cells as a potential target for treatment options, and the advantages of primary cultures over standard cell lines, it is necessary to clarify and validate methods for culturing neurospheres from primary material in neuroblastoma.

It is therefore important to investigate avenues of utilizing shorter term primary cultures such as those established in stem cell media, based on the hypothesis that they may still remain representative of the driver genetic features of the original tumor stem cells, and have limited adaptive changes to tissue culturing conditions. Hence, we sought to establish neurosphere primary lines from neuroblastoma surgical samples, to determine the reproducibility of the technique, and to generate a resource for future research studies in therapeutics, genetics and stem cell biology. We found a high overall success rate (55%) of establishment from surgical samples.

## Methods

### Patients and consent

The study had ethical review board approval (REC reference 14/WM/1253 “Establishing primary cultures and cell lines from pediatric cancers”) and samples were made available following informed consent. Patients were eligible if there was a known or suspected diagnosis of neuroblastoma. All samples were included based on known or suspected diagnosis of neuroblastoma without selection on clinical criteria. In some cases with insufficient material for culture, the transport fluid used for sample transfer (0.9% saline) was placed directly into culture.

### Tumor preparation

Tru-cut needle biopsies or tissue from tumor resections from neuroblastoma patients were transferred directly from operating theatre to the hospital histopathology laboratory. Following sterile cut up and routine diagnostic processing including freezing of material for research, surplus material was evaluated by a consultant pathologist for tissue viability and made available for culture. Tissue was manually disaggregated in a 10cm tissue culture dish using a sterile scalpel. Tumors that dissociated readily in this way were placed immediately into stem cell media in 25cm ^2^ flasks, 24 well plates or 12 well plates depending on the available tissue. Since it is not possible to perform an accurate cell count on the partially dissociated tumor, choice of culture container was based on estimation of size that would yield approximately 100% confluence, were the sample fully disaggregated. Where spare solid material was unavailable from a biopsy, up to 1ml of saline used to transport the sample to the histopathology laboratory was taken and added to 4ml of stem cell media in a 25cm ^2^ flask. When more than 1ml of transit fluid was provided, it was centrifuged at 300G for 5 minutes and the pellet resuspended in stem cell media. Samples that were tougher to disaggregate were digested with Accutase (Thermofisher 00-4555-56) for up to 1 hour, or until obvious disaggregation was observed, and divided between wells of a multiwell plate based on the estimation of confluence as above.

### Culture conditions

Disaggregated cells and tumor fragments were placed into standard serum free neurosphere culture media (Stem Cell Media, SCM) composed of DMEM/F12 (Sigma-Aldrich D8437),° 1% B27 (Gibco 17504044) with 20ng/ml EGF (Sigma Aldrich #E9644) and 20ng/ml FGF (Fibroblast Growth Factor, Peprotech 100 – 18B) in the presence of penicillin and streptomycin antibiotics. Cells were propagated at 37°C in 5% CO _2_. Cultures were inspected twice a week for cell density and presence of neurospheres, and split 1:2 to 1:5 depending on cell density and speed of growth. Cultures were frozen in serum free DMEM/F12 media with 10% DMSO at approximately 10 ^7^ cells per ml.

### Neurosphere disaggregation and flow cytometry

A total of 14 randomly selected representative primary cultures were analyzed by flow cytometry. To produce a single cell suspension necessary for this, cells growing as a monolayer were detached with Accutase (Thermofisher) according to the manufacturer’s instruction, while neurospheres were disaggregated with Accutase and the mechanical force of gentle pipetting. Surviving cells, re-suspended into single cell suspension, were stained in FACS tubes using saturating amounts (1–5ul of stock) of antibody in 100ul PBS using the following panel of directly conjugated monoclonal antibodies all from Biolegend: GD2-PE (357304, RRID: AB_2561885), CD56-APC/Cy7 (318332, RRID: AB_10896424), CD45-BV711 (304049, RRID: AB_2563465) controlled for nonspecific staining using isotype-matched labelled polyclonal antibodies. The stained primary culture cells were incubated on ice with antibody mix for 30 minutes and then washed with PBS and centrifuged at 300G for 5 minutes. Data was collected with the BD LSRII flow cytometer (BD Biosciences) and data analysis used FlowJo software (v8.8.3).

The gating strategy excluded dead cells by a live/dead stain using DAPI. To exclude leukocytes that may be present from blood in the original sample, the resulting live population was gated on CD45 negative and CD56 positive cells, the latter a standard marker for neuroblastoma. This gated cell population of CD45-ve/CD56+ve cells was then examined for expression of GD2, also ubiquitously expressed on neuroblastoma.

### Statistical analysis

Chi-squared tests were used to determine significance of association of categorical variables; for example successful versus non successful expansion correlated with pre versus post chemotherapy. Data were tabulated and tests of significance were performed using Microsoft Excel 2016 and GraphPad Prism v6.0

## Results

### Neurosphere cultures can be established from the majority of neuroblastoma patient samples grown in suspension cultures and retain typical neuroblastoma surface immunophenotype

Between October 2014 to end of 2016, tumor biopsies or resections of 67 consecutive neuroblastoma samples from 52 patients treated at Great Ormond Street Hospital London were systematically evaluated for their ability to establish primary neurosphere lines. Of these, 39 were primary needle biopsies and 28 were surgical resections of which 24 were post chemotherapy resections and 4 were primary (treatment-naïve) resections.

The success in terms of establishment of cultures was determined by the ability of cultures to produce discrete visible neurospheres. All cultures derived from neuroblastoma samples were observed to grow as a mixture of phenotypes including neurospheres, single suspension cells, and as monolayer, although often one of the three growth patterns was predominant. Cases could be dichotomized into those that formed spheres within the first 14 days of establishment and then went on to form long term successful cultures, and those that never formed spheres and were classified as “unsuccessful cultures”. The overall success rate, as thus defined, was 55%. It appeared that successful establishment of neurospheres could be made from chemotherapy-naïve primary tumors and post chemotherapy surgical samples, as well as primary needle biopsies. Successful establishment of lines is summarized in Table 1. Of 43 chemotherapy-naive samples (39 biopsies and 4 surgical excisions), 29 (67%) were successfully established, whereas for the post-chemotherapy surgical samples success rate was 8 out of 24 (33%), which is significantly inferior (Chi Sq p=<0.008) (see underlying data ^8^). This suggests that the protocol, if applied to optimally procured tissue, would have a success rate over 65%.

**Table 1.** Establishment of sphere cultures from primary sample type. Of the chemotherapy-naïve surgical excisions, 2 of these 4 were from recurrences.

	Samples	Sphere cultures (%)
**Total samples**	**67**	**37 (55)**
Total fluid only samples	17	9 (53)
Total solid samples	50	28 (56)
**Total biopsy samples**	**39**	**25 (64)**
Diagnostic biopsies	34	21 (62)
Recurrence biopsies	5	4 (80)
Biopsy fluid only cultures	15	9 (60)
**Total excision samples**	**28**	**12 (43)**
Post chemotherapy	24	8 (33)
Chemotherapy naive	4	4 (100)

Due to the impossibility of counting cells both at the start of culturing and following establishment of spheres, it is not possible to plot growth curves. However, the range of time from initial seeding to establishment of a confluent 75cm ^2^ flask is approximately 2 to 10 weeks showing the marked heterogeneity of growth rates.

**Figure 1.**
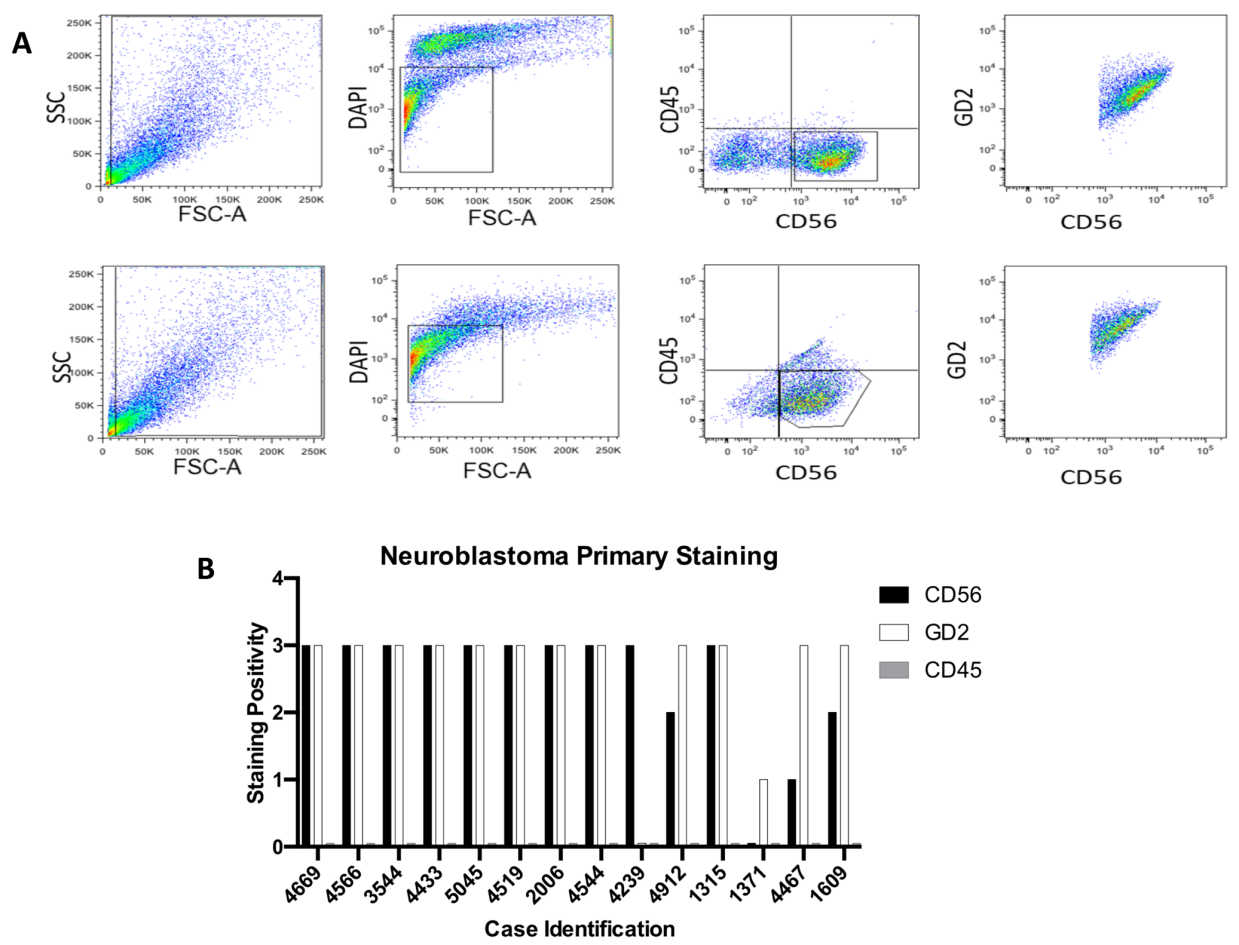
Neurospheres derived from neuroblastoma primary tumors retain typical immunophenotype. **A**) representative flow cytometric staining for two independent cultures (excision culture 3544 (upper) and biopsy culture 4669 (lower) following gating on live cells. **B**) graphical representation of the staining pattern in 14 evaluated samples; positivity was categorized into dim, medium and bright based on MFI and scored as 1, 2 and 3 respectively.

In order to determine that these were neuroblastoma cells, 14 representative samples were analyzed by flow cytometry. Cells from primary cultures were assessed for expression of the pan leucocyte marker CD45, which neuroblastoma cells do not express, and for ubiquitously expressed NBL markers CD56 and GD2. Of the samples tested, 79% exhibited this characteristic neuroblastoma staining pattern. We found that the neurosphere cultures showed a very bright and homogeneous staining for both NBL markers ( Figure 1)

### Neurosphere cultures can be successfully established from transit fluid samples and retain typical neuroblastoma immunophenotype

We were interested in whether it is possible to establish neuroblastoma lines when using very small amounts of tissue. In cases with insufficient tissue to culture, 1ml of transportation fluid was put directly into 4ml stem cell media (SCM) in a 25cm ^2 ^tissue culture flask, testing the hypothesis that small amounts of residual tumor cells within the transport media would be able to establish. Where transport fluid provided was more than 1ml, this was centrifuged and the pellet resuspended in SCM. Cultures established from transit fluid had a comparable success rate to those established from solid tissue samples (9 out of 15= 55% versus 25 out of 39 =64% for biopsies: Chi Sq p=0.78).

To perform a further unbiased comparison, eight patients had neurospheres cultured from the same biopsy with separate culturing of the solid tissue and surgical transit fluid. In three there was no successful growth, in three there was concordance of growth from the two sources, and in two there was success from solid tissue but not transit fluid; there were no cases with success of transit fluid but failure from solid tissue. Therefore transit fluid appears a less reliable source of material but a useful adjunct in the context of scarce material.

### Heterogeneity of *in vitro* growth phenotype and adaptation to growth on laminin

In addition to growth as neurospheres, primary cultures established in SCM were observed to grow as adherent monolayers, single cell suspensions, or mixtures of all three growth patterns. Moreover, there is marked heterogeneity in the size of neuropheres both within and between samples. It is not possible to quantify this heterogeneity, but all the NBL SCM lines contained some elements of adherent, single cell suspension and neurospheres. SCM derived from gliomas can be grown as adherent cultures by coating culture flasks with laminin and so we were interested in whether neuroblastoma primary cultures could also adopt this phenotype. We did not attempt initial establishment in laminin in any cases. In our hands, when established or establishing SCM lines were transferred to laminin, the degree of attachment increased markedly, with outgrowth of adherent spheres and associated adherent cells growing out as a monolayer. In some cases where growth in suspension appeared slow, the change to laminin appeared to accelerate growth. Because of the impossibility of counting cells in neuropheres without majorly disrupting them, it was not possibly to quantify these aspects of altered growth in laminin. On return of laminin cultured cells to non-coated flasks, they reverted to predominant suspension growth pattern.

### No significant association between success rate with clinical/histological features

Patient characteristics and ability to establish neurospheres is shown in Table 2. The number of male samples were 37 out of 67. The ability to establish neurospheres did not vary depending on gender (59% vs 50%). Biopsies which demonstrated only undifferentiated neuroblastoma had a higher but non-significant success rate in establishing neurospheres than those which showed any evidence of differentiation or post chemotherapy changes (60% vs 45%). Age, stage, MYCN amplification and the presence of segmental chromosomal aberrations were all non significant in terms of neurosphere success rate ( Table 2). 

**Table 2.** Patient features and correlation with establishment of spheres. There were no significant associations with neurosphere establishment (Chi-square). L1, L2, M or MS stage disease at diagnosis used the International Neuroblastoma Risk Group (INRG) Classification System.

	Number (%)	Successful Establishment of spheres (%)
**Gender**		
Male	37 (55)	22 (59)
Female	30 (45)	15 (50)
**Age**		
<=18months	36 (54)	23 (64)
>18months	31 (46)	14 (45)
**INRG Stage at Diagnosis**		
L1	3 (4)	2 (67)
L2	21 (31)	14 (67)
M	39 (58)	18 (46)
MS	4 (6)	3 (75)
**NMYC Amplification**		
Yes	17 (25)	7 (41)
No	50 (75)	30 (60)
**Segmental Chromosomal Abnormalities**			
Yes	21 (31)	14 (67)
No	46 (69)	23 (50)
**Tumor Biology**		
Undifferentiated	47 (70)	28 (60)
Differentiating	20 (30)	9 (45)

Six patients had two samples collected at different time points during treatment. For four of them there was no concordance for success between the timepoints whereas two patients (one success and one failure) the different timepoints were concordant. Therefore we failed to find any evidence that patient-specific factors govern success rate. 

## Discussion

Here we present data from a series of 67 samples diagnosed with neuroblastoma, from which tumor tissue or surgical transit fluid was obtained and used to culture tumor spheres under conditions commonly used in the culture of neural stem cells ^6^. Tumor spheres were subsequently dissociated and assessed by flow cytometry for presence of neuroblastoma markers GD2 and CD56, to show that cells and tumor spheres grown in culture retain the characteristic markers the cells of their tissue of origin. These cells may then be used as targets *in vitro* for drug development, or for engraftment *in vivo* for the development of patient-derived xenograft models that better reflect the heterogeneity of this disease.

Although well established that it is possible to use SCM to propagate stem cells in the form of neurospheres from a number of different cancer types ^9^, the literature is sparse in regards to their evaluation in neuroblastoma. We have therefore systematically evaluated success rate and shown that with simple culturing techniques it is possible to establish primary lines which retain the characteristic immunophenotypic features of neuroblastic tumors. Further studies will be required to establish the success rate of formation of tumors, for example in xenograft models. Moreover, it will be interesting to study the ability of these cells to differentiate into neuroblasts *in vitro,* for example by transfer to normal growth media with serum, as well as the mutational drift compared to the original tumor if any.

It would be advantageous to have a method of establishment of primary cell culture which reproducibly leads to cell expansion. Even if the expanded cells represent a subclone that are adapted to *in vitro* conditions (for example cancer stem cell expansion in stem cell media), this still represents an advantage over methods such as establishment of cell lines in normal growth media with serum, that typically go through a crisis phase before establishment of an emergent line that is presumably highly evolved for adaptation to tissue culture conditions, and therefore less likely to represent the *in vivo* tumor. Conventional two-dimensional monolayer models have been useful in understanding many of the characteristics of neuroblastoma cell biology, but in addition to the concerns about genetic evolution to adapt to typical cell culture conditions, they do not accurately reflect the multicellular physiology of tumors *in vivo* or how the tumor microenvironment is developed. Neither adherent monolayer cultures nor neurospheres are physiological, and occur only during *in vitro* culturing conditions, however the latter may offer some advantages as a research tool. Although neurospheres do not have the architecture and vasculature of *in vivo* tumors, their three-dimensional structure may have advantages of cell monolayers in terms of better representing aspects of three-dimensional tumors such as central hypoxia and drug penetrance. Future studies on neurosphere permeability and response to therapeutics might be of interest to evaluate their role as an *in vitro* tumor model.

The establishment of neurospheres comprised of primary neural stem cells has also been frequently used and there exist a variety of methods for their establishment ^6^. However, the sphere culture model is limited by its sensitivity to culture technique including sensitivity to alterations in media or cell density, or frequent passaging and dissociation ^5, 10^. In our hands the neuroblastoma SCM cultures do show this sensitivity to changes in environmental conditions but retain the capacity to recover from stress if left alone in normal SCM growth conditions. Due to the long-term nature of their propagation, meticulous attention to sterility is needed to prevent the outbreak of microbial contamination. 

While neurospheres represent a straightforward approach for maintaining cancer cells in culture, and whilst they are a potentially useful tool for cancer stem cell investigations, further studies are needed to determine how useful they will be in developing biological or therapeutics models for basic or translational research. Our demonstration of high success rate of their establishment using a uniformly applied protocol is a valuable contribution to the field for identifying an alternate and reproducible approach to expanding the reagents available for neuroblastoma research.

## Ethics approval and consent to participate

The study had ethical review board approval (REC reference 14/WM/1253 “Establishing primary cultures and cell lines from pediatric cancers”) and all patient samples were collected with written full consent

## Data availability

### Underlying data

Open Science Framework: Primary neuroblastoma cultures database. https://doi.org/10.17605/OSF.IO/T2RFB ^8^

This project contains the following underlying data:

- FACS data (folder containing output FACS files for different cases)
- Flow Cases - reference table.docx (Reference table for FACS output files)
- Full data sheet no identifiers.xlsx (spreadsheet of sample characteristics)

Data are available under the terms of the Creative Commons Attribution 4.0 International license (CC-BY 4.0).
