# Supplementary material for: Establishment and phenotyping of neurosphere cultures from primary neuroblastoma samples
Source: F1000Res. 2019 Jun 10;8:823. [Version 1] doi: 10.12688/f1000research.18209.1 (PMC6611133; doi:10.12688/f1000research.18209.1)
Supplement: Peer review history

# Peer review: Reviewer response for version 1

**DOI:** 10.5256/f1000research.19917.r49684

**Published:** 2019-07-08

**Authors:** Gudrun Schleiermacher

**Affiliations:** 1 SIREDO Department of Pediatric Oncology, Institut Curie, Paris, France; 2 INSERM U830, RTOP (Recherche Translationelle en Oncologie Pediatrique), Institut Curie, Paris, France

Neuroblastoma is a clinically and genetically heterogeneous pediatric tumor. Precise biological and molecular characterization of tumor cells is increasingly becoming an integral part of patient care, with treatment stratification depending not only on clinical, but also on molecular characteristics. However, tumor samples are frequently small, especially when obtained by fine needle biopsies, with frequently only a limited amount of biological material available for increasingly complex molecular investigations or cellular screening procedures.

 In this manuscript, the authors describe the technical aspects of direct culture of neuroblastoma samples for the establishment of neurosphere cultures. Importantly, they describe a procedure which is applied to consecutive cases in a single center, with samples obtained by different techniques (surgical resections, fine needle biopsies, transport fluid) and at different time points (at diagnosis, post chemotherapy). The success rate is 55% (37/67 samples), without any significant association between the success rate of establishing a neurosphere culture and clinical/histological/genetic features. Characterization of the obtained neurosphere cultures is still limited, but the results indicate that the cultured cells do express neuroblastoma markers.

 The ultimate usefulness of the established neurosphere cultures will depend on a number of aspects. It will be necessary to compare genetic/epigenetic/cell identity features between the primary tumor and corresponding neurosphere culture, to search more in detail for features linked to culture success rates and to establish the duration of tumor growth.

 The importance of this work is linked to the presentation of a reproducible method for the expansion of frequently rare tumor tissue, enabling more large scale use of such samples for molecular characterization, drug screening and other applications for the patients’ benefit.

I confirm that I have read this submission and believe that I have an appropriate level of expertise to confirm that it is of an acceptable scientific standard.

# Peer review: Reviewer response for version 1

**DOI:** 10.5256/f1000research.19917.r49683

**Published:** 2019-07-02

**Authors:** Juliet C. Gray

**Affiliations:** 1 Antibody and Vaccine Group, Centre for Cancer Immunology, Faculty of Medicine, Department of Paediatric Oncology, University of Southampton, Southampton, UK

Neuroblastoma is a rare paediatric tumour which accounts for a disproportionately high number of paediatric cancer deaths. Fresh tissue samples are usually small and scarce, and established cell lines are likely to poorly represent the heterogenous nature of the disease. This paper describes a simple methodology for establishing neurosphere cultures from small samples of fresh biopsy tissue, with a relatively high success rate. This is a potentially very useful technique for extending the use of limited primary tissue samples to maximal benefit. Of note, success was even achieved with fluid  from around surgical/biopsy samples, rather than solid tissue samples, which is potentially very useful when sample size is limited.

 The article is clearly written and will be of interest to those working in the field.

I confirm that I have read this submission and believe that I have an appropriate level of expertise to confirm that it is of an acceptable scientific standard.

# Peer review: Reviewer response for version 1

**DOI:** 10.5256/f1000research.19917.r49685

**Published:** 2019-07-01

**Authors:** Toby Trahair

**Affiliations:** 1 Kids Cancer Centre, Sydney Children's Hospital, Randwick, NSW, Australia; 2 Children's Cancer Institute, Lowy Cancer Research Centre, UNSW, Kensington, NSW, Australia; 3 School of Women's and Children's Health, UNSW Medicine, NSW, Australia

This is a straightforward technical report on direct culture of neuroblastoma samples from consecutive cases treated at Great Ormond Street.

 The research team has taken a logical approach to establishing an easy method to establish patient-derived neurospheres. Tumour samples were derived from a number of sources (open biopsy, needle biopsy & from transport fluid). The overall success rate (37 cultures from 67 samples) confirms that this approach is feasible.

 The characterisation of the resulting neurosphere cultures has been limited, but expression of both CD56 and GD2 suggests that the cultures really are neuroblastic tumours.

 The significance of this work really lies in the downstream application of patient-derived cultures. There are a number of unanswered questions, specifically how representative the neurosphere cultures are of the donor tumour, whether there is any correlation between the capacity to establish a culture and patient outcome, whether the neurosphere models can be propagated as xenografts, and if it is possible to derive neurosphere cultures from each genetic/genomic subtype of neuroblastoma (e.g. MYCN amplified, TERT rearranged, ATRX deleted, etc.).

 Overall, with an increasing focus on personalisation of therapy, whilst a simple piece of work, I believe it is a valuable contribution to the field and will be helpful for other groups engaged in such work.

I confirm that I have read this submission and believe that I have an appropriate level of expertise to confirm that it is of an acceptable scientific standard.

# Peer review: Reviewer response for version 1

**DOI:** 10.5256/f1000research.19917.r49686

**Published:** 2019-07-01

**Authors:** Dermot Murphy

**Affiliations:** 1 Department of Oncology, Royal Hospital for Sick Children, Glasgow, UK

The article is scientifically sound. My only criticism is stylistic. I think the sentences are too long and the punctuation could be better. The text is clunky.

- For example:
  “The disease is strikingly heterogeneous with a range of clinical phenotypes, from spontaneous regression, including in cases with metastases, to aggressive disease with marked resistance to radiotherapy and chemotherapy. New approaches to therapies supported by the availability of suitable tumor models that more closely resemble the human disease are essential for improving outcomes for this disease.”
- Edit to:
  Neuroblastoma is strikingly heterogeneous. Clinical phenotypes range from spontaneous regression to aggressive disease characterised by marked resistance to radio and chemotherapy. New therapeutic approaches, supported by tumor models more closely resembling human disease, are essential for improving outcomes in children.

There are many more examples in the subsequent manuscript. Tight editing would vastly improve readability.

I confirm that I have read this submission and believe that I have an appropriate level of expertise to confirm that it is of an acceptable scientific standard.
